# Deaths Associated with Human Adenovirus-14p1 Infections, Europe, 2009–2010

**DOI:** 10.3201/1708.101760

**Published:** 2011-08

**Authors:** Michael J. Carr, Adriana E. Kajon, Xiaoyan Lu, Linda Dunford, Paul O’Reilly, Paul Holder, Cillian F. De Gascun, Suzie Coughlan, Jeff Connell, Dean D. Erdman, William W. Hall

**Affiliations:** Author affiliations: National Virus Reference Laboratory–University College Dublin, Dublin, Ireland (M.J. Carr, L. Dunford, P. O’Reilly, P. Holder, C.F. De Gascun, S. Coughlan, J. Connell, W.W. Hall);; Lovelace Respiratory Research Institute, Albuquerque, New Mexico, USA (A.E. Kajon);; Centers for Disease Control and Prevention, Atlanta, Georgia, USA (X. Lu, D.D. Erdman)

**Keywords:** Adenovirus serotype 14, HAdV-14p1, molecular epidemiology, phylogenetics, restriction enzyme analysis, immunocompromised, immunocompetent, ARD, fatalities, viruses, Europe, research

## [MEDSCAPECME LOGO]

Medscape, LLC is pleased to provide online continuing medical education (CME) for this journal article, allowing clinicians the opportunity to earn CME credit.

This activity has been planned and implemented in accordance with the Essential Areas and policies of the Accreditation Council for Continuing Medical Education through the joint sponsorship of Medscape, LLC and Emerging Infectious Diseases. Medscape, LLC is accredited by the ACCME to provide continuing medical education for physicians.

Medscape, LLC designates this Journal-based CME activity for a maximum of 1 *AMA PRA Category 1 Credit(s)*^TM^. Physicians should claim only the credit commensurate with the extent of their participation in the activity.

All other clinicians completing this activity will be issued a certificate of participation. To participate in this journal CME activity: (1) review the learning objectives and author disclosures; (2) study the education content; (3) take the post-test with a 70% minimum passing score and complete the evaluation at www.medscape.org/journal/eid; (4) view/print certificate.

**Release date: July 22, 2011; Expiration date: July 22, 2012**

## Learning Objectives

Upon completion of this activity, participants will be able to:

- Describe demographic, genetic, and transmission-related factors associated with human adenovirus-14p1 (HAdV-14p1) infections recently detected in Ireland
- Describe clinical characteristics and complications associated with HAdV-14p1 infections recently detected in Ireland
- Describe the clinical and public health implications of these findings.

## MedscapeCME Editor

**Karen Foster, **Technical Writer/Editor, *Emerging Infectious Diseases. Disclosure: Karen Foster has disclosed no relevant financial relationships.*

## MedscapeCME Author

**Laurie Barclay, MD, **freelance writer and reviewer, Medscape, LLC. *Disclosure: Laurie Barclay, MD, has disclosed no relevant financial relationships.*

## Authors

*Disclosures:* ***Michael J. Carr, PhD; Adriana E. Kajon, PhD; Xiaoyan Lu, MS; Linda Dunford, MSc; Paul O’Reilly, BSc; Paul Holder, MSc; Cillian F. De Gascun, MD, FRCPath; Suzie Coughlan, PhD; Jeff Connell, PhD, FRCPath; Dean D. Erdman, DrPH;*** *and* ***William W. Hall, MD, PhD,*** *have disclosed no relevant financial relationships.*
