# Supplementary material for: Deaths Associated with Human Adenovirus-14p1 Infections, Europe, 2009–2010
Source: Emerg Infect Dis. 2011 Aug;17(8):1402–8. doi: 10.3201/1708.101760 (PMC3381588; doi:10.3201/1708.101760)
Supplement: Related texts

# Research article: Deaths Associated with Human Adenovirus-14p1 Infections, Europe, 2009–2010

**DOI:** 10.3201/1708.101760

**Authors:** Michael J. Carr, Adriana E. Kajon, Xiaoyan Lu, Linda Dunford, Paul O’Reilly, Paul Holder, Cillian F. De Gascun, Suzie Coughlan, Jeff Connell, Dean D. Erdman, William W. Hall

**Affiliations:** Author affiliations: National Virus Reference Laboratory–University College Dublin, Dublin, Ireland (M.J. Carr, L. Dunford, P. O’Reilly, P. Holder, C.F. De Gascun, S. Coughlan, J. Connell, W.W. Hall);; Lovelace Respiratory Research Institute, Albuquerque, New Mexico, USA (A.E. Kajon);; Centers for Disease Control and Prevention, Atlanta, Georgia, USA (X. Lu, D.D. Erdman)

## Abstract

Human adenovirus (HAdV) serotype 14 is rarely identified. However, an emerging variant, termed HAdV-14p1, recently has been described in the United States in association with outbreaks of acute respiratory disease with high rates of illness and death. We retrospectively analyzed specimens confirmed positive for HAdV by immunofluorescence, virus culture, or real-time PCR during July 1, 2009–July 31, 2010, and describe 9 cases of HAdV-14p1 infection with characteristic mutations in the fiber and E1A genes that are phylogenetically indistinguishable from the viruses previously detected in the United States. Three patients died; 2 were immunocompromised, and 1 was an immunocompetent adult. We propose that surveillance should be increased for HAdV-14p1 and recommend that this virus be considered in the differential diagnosis of sudden-onset acute respiratory disease, particularly fatal infections, for which an etiology is not clear.

Human adenoviruses (HAdVs) were identified independently by 2 groups during the early 1950s (*1**,**2*). HAdVs are nonenveloped, linear double-stranded DNA viruses encapsidated within a protein shell and have been categorized into 6 species (A–F) that contain 51 immunologically distinct serotypes (*3*). HAdVs most commonly cause acute respiratory disease; however, depending on the infecting HAdV serotype and tropism resulting from differential host receptor use, the wide variety of symptoms can include pneumonia, febrile upper respiratory illness, conjunctivitis, cystitis, and gastroenteritis (*4*). The severity of disease appears dependent on the immunocompetence and cardiopulmonary health of the host, and the spectrum of disease can range from subclinical to severe respiratory distress and death (*4*). Immunocompromised patients (especially bone marrow transplant [BMT] recipients) are particularly susceptible to HAdV infection, resulting in severe illness and deaths, whereas illness in immunocompetent patients generally resolves without major complication.

HAdV species B comprises 2 subspecies: B1 (including HAdV-3, -7, -16, -21, and -50) and B2 (HAdV-11, -14, -34, and -35). The subspecies B2 HAdV-14 (agent de Wit) was originally identified as an etiologic agent of acute respiratory disease in military recruits in the Netherlands during 1955 (*5*). Despite reports of subsequent outbreaks in Europe and Asia during the 1950s and early 1960s (*6*), global surveillance in the subsequent decades had not identified circulation of this serotype until spring 2006, when HAdV-14 emerged as a cause of a major proportion of acute febrile respiratory illness in military bases across the United States (*7**–**9*). In 2007, community-associated outbreaks were identified in California and New York (*10*), and during March–June 2008, ≈140 cases of HAdV-14 were identified in Oregon, Washington, and Texas. Overall, 38% of patients were hospitalized, 17% were admitted to intensive care units, and 5% died. During September 2008, an outbreak was reported with 32 cases of pneumonia on Prince of Wales Island off the coast of Alaska (*11*). In the United States, the earliest documented case of HAdV-14 infection, by retrospective testing, occurred in California during December 2003, and the most recent evidence of HAdV-14 had been in Pennsylvania during June 2009 (*7*). The virus has circulated uninterrupted in some military recruit camps (A.E. Kajon, unpub. data).

Sequence analysis of the fiber gene of HAdV-14 associated with the recent outbreaks has shown a 6-bp deletion resulting in a 2-aa deletion (lys-glu) at positions 251 and 252 in the knob region compared with the de Wit HAdV-14p prototype strain (*10*). The 252glu site is conserved in all other species B HAdVs and is located in the F–G loop of the fiber protein knob near a putative host receptor binding site. Further sequencing of the hexon gene hypervariable regions 1–7 and the E1A genes demonstrated that this emerging HAdV-14 was clearly separable from the reference strain de Wit (genome restriction type HAdV-14p) (*9**,**11*). This new genomic variant has been designated HAdV-14p1 on the basis of novel restriction profiles. Viral receptor binding and internalization studies that used recombinant HAdV-14p1 fiber regions containing the 2-aa deletion did not demonstrate substantial phenotypic differences between the new agent and the prototype virus (*12*). This finding suggests that HAdV-14p1 could be an immune escape mutant that has lost a potential neutralizing epitope, has modified postinternalization steps, or has enhanced binding to host cells through a yet-unidentified viral receptor.

However, no evidence suggests that HAdV-14p1 has reemerged because of altered virulence. Despite the mutations in the fiber and EIA genes, no other genetic differences with the prototype HAdV-14 clearly explain differing disease severity in US outbreaks (*13*). This observation suggests that reemergence of this agent might be more likely to have resulted from spread of an infectious agent in immunologically naive populations and changes in the rate of specific immunity in host populations over time. The recent whole-genome analysis of HAdV-14p1 in mild and severe infections supports this idea (*13*). In addition, reports of recent HAdV-14 and HAdV-14–11 outbreaks in Asia and the United States have detailed similar dynamics (*14**,**15*). The observed characteristics (including periods of little activity punctuated by distinct outbreaks with occasional severe disease and death) may simply be the natural pattern of adenovirus species B2 respiratory pathogens.

Whether HAdV-14p1 had circulated elsewhere before, or at the same time as, the outbreaks in the United States and whether it is currently circulating in Europe is unclear. In this report, we show that recent infection and deaths associated with HAdV-14p1 infection have occurred in Ireland and that this virus is genetically indistinguishable from HAdV-14p1 described in the United States.

## Materials and Methods

### Study Period

The study period was July 1, 2009–July 31, 2010. The study comprised 29 cases confirmed adenovirus positive by indirect immunofluorescence assay (IFA), viral culture, or real-time quantitative PCR (qPCR) at the National Virus Reference Laboratory (NVRL, Dublin, Ireland).

### Virus Culture and Restriction Enzyme Analysis

HAdVs were cultured at NVRL and Lovelace Respiratory Research Institute (Albuquerque, NM, USA). Isolates were passaged once in human embryonic lung carcinoma cells in 25-cm^2^ flasks for detection of cytopathic effects and then subsequently expanded in 75-cm^2^ flasks for extraction of viral DNA for restriction enzyme analysis as described (*16*). Viral DNA was digested with the same panel of endonucleases used to characterize the North American strain of HAdV-14 (*7*).

### Indirect Immunofluorescence

We performed IFA for influenza A virus, influenza B virus, parainfluenza viruses 1–3, respiratory syncytial virus, and HAdV. We used the respiratory virus panel kit (Biotrin, Dublin, Ireland) and followed the manufacturer’s instructions.

### Molecular Analysis

We performed qPCR for HAdV as described (*17*). Adenoviruses were initially typed by using partial hexon gene sequencing as described (*17**,**18*). We extracted 200 µL of serum, plasma, or nasopharyngeal aspirate by using the QIAamp DNA Mini Kit (QIAGEN, Crawley, UK) according to the manufacturer’s instructions or by using the MagNA Pure automated extraction platform (Roche, Lewes, UK) with an external lysis step. The final elution volumes for both methods was 50 µL. Real-time PCR specific for HAdV-11 and HAdV-14 (*19*) provided by the Centers for Disease Control and Prevention (Atlanta, GA, USA) was performed by using the ABI7500 SDS platform (Applied Biosystems, Foster City, CA, USA) with 5 µL of template and the Platinum qPCR SuperMix-UDG kit (Invitrogen, Paisley, UK) with 0.5-µmol/L forward and reverse primers and 0.1-µmol/L probe labeled at the 5′ end with 6-carboxyfluorescein and a 3′ quencher dye in 25-µL single-plex assays performed with the following cycling conditions: 50°C for 2 min, then 95°C for 2 min, followed by 45 cycles of 95°C for 15 sec and 60°C for 1 min, with data acquisition in the anneal/extension phase. Complete HAdV-14 fiber, E1A, and hexon genes were amplified as described (*7*) and sequenced bidirectionally. Nucleotide sequence data for HAdV-14p1 fiber, E1A, and hexon genes were submitted to GenBank (accession nos. HQ163915, HQ163916, and HQ265808).

### Phylogenetic Analysis

The fiber, E1A, and hexon genes of the Ireland HAdV-14p1 strains were compared with recently described US HAdV-14p1 strains (*7*) and reference sequences from HAdV B2 subgenera obtained from GenBank. The accession numbers for all sequences are included on the tree (Figure 1). Lasergene version 8 (DNASTAR, Madison, WI, USA) was used for contiguous assembly (*20*), and the sequences were aligned by using ClustalW (*21*) implemented in Bioedit version 7.05 (*22*). Phylogenetic trees were constructed by using the maximum-likelihood method under the HKY85 substitution model that used PAUP* version 4.0 β 10 (*23*), and bootstrapping with 1,000 replicates was used to analyze the stability of the tree topology.

**Figure 1:** Maximum-likelihood trees of the full-length fiber (A), E1A (B), and hexon (C) open reading frames of adenovirus B2 subgenera. Phylogenetic analysis was performed by using reference sequences from GenBank for the adenovirus B2 subgenera, including prototype reference strains. The query sequences from this study are identical and are represented in **boldface**. The tree was built in PAUP* (*23*) on the basis of the HKY85 model of evolution and for the fiber tree also with a β distribution and used midpoint rooting. Bootstrap resampling (n = 1,000) was performed by using the neighbor-joining algorithm. Scale bars indicate nucleotide substitutions per site.

## Results

We retrospectively tested clinical specimens received by NVRL from 29 patients with positive results for HAdV by IF, virus culture, or qPCR during the study period by using specific HAdV-11 and -14 real-time assays. All 29 samples were negative for HAdV-11; however, 9 (31%) samples tested positive for HAdV-14 (Table). All patients sought care or were previously hospitalized during November 2009–July 2010. Of the 9 patients, 7 (78%) were male, 6 (67%) patients were <4 years of age, and the remaining 3 patients were >34 years of age. No epidemiologic links were known between any of the patients. No geographic clustering was observed; the cases were distributed throughout Ireland and involved several hospitals. All respiratory samples were IFA negative for the following respiratory viruses: influenza A, influenza B, parainfluenza viruses 1–3, and respiratory syncytial virus. Our earliest identified HAdV-14–positive specimen (November 10, 2009; case-patient 1 in Table) was positive for influenza A virus by real-time reverse transcription PCR and confirmed as pandemic influenza A (H1N1) 2009 on October 29. No influenza A was detected in a specimen from this patient on November 8 and a nasopharyngeal aspirate collected 2 days later; however, this later specimen was positive for HAdV-14.

**Table:** Clinical characteristics of patients with confirmed HAdV-14p1, Ireland, July 1, 2009–July 31, 2010*

Case no.	Age/sex	Location	Sample date	Sample type	Previous condition	Clinical characteristics	Outcome
1	4 y/M	Dublin	2009 Nov	NPA	Pierre Robin syndrome (craniofacial abnormality)	Fever, tachypnea, cough	Survived
2	7 mo/M	Dublin	2010 Mar	NPA	None known	Bronchiolitis	Survived
3	1 mo/M	Dublin	2010 May	NPA	None known	Bronchiolitis, diarrhea, vomiting	Survived
4	8 d/F	Dublin	2010 May	Serum (×2), urine	Preterm birth at 35 weeks’ gestation	Hypotonic, abnormal LFT results	Died
5	46 y/F	Kilkenny	2010 May	Other/lung biopsy	Smoking, high BMI	Community-acquired pneumonia, sepsis	Died
6	34 y/M	Dublin	2010 May	BAL	Post-BMT, neutropenic	Unilateral pulmonary infiltrate, fever	Died
7	4 mo/M	Dublin	2010 Jul	NPA	None known	Bronchiolitis, diarrhea, vomiting	Survived
8	14 d/M	Cork	2010 Jul	Plasma (×2)	None known	ARDS	Survived
9	48 y/M	Dublin	2010 Jul	BAL	HIV+ (RNA <50) HCV+ (RNA not detected); CD4 436 (15%)	Pneumonia	Survived

*HAdV-14p1, human adenovirus serotype 14p1; NPA, nasopharyngeal aspirate; LFT, liver function test; BMI, body mass index; BAL, bronchoalveolar lavage; BMT, bone marrow transplant; ARDS, acute respiratory distress syndrome; +, positive; HCV, hepatitis C virus; CD4, CD4 cells/mL.

Fiber, E1A, and hexon gene sequences derived from the 9 HAdV-14 case-patients from Ireland during the study period were 100% identical for each gene analyzed. These sequences were then compared with subspecies B2 HAdV reference sequences from GenBank, including the prototype de Wit strain, HAdV-14p (*5*). Phylogenetic analysis demonstrated that the Ireland and US HAdV-14p1 fiber, E1A, and hexon sequences formed a monophyletic group (Figure 1, panels A–C). The sequences from Ireland were 100% identical in the fiber, E1A, and hexon open reading frames (ORFs) to the recently described HAdV-14p1 strains isolated in the United States during 2003–2009 (*7**–**9*).The fiber ORF sequences analyzed from the isolates from Ireland were 972 nt, and all contained the 6-nt deletion identified in the recent US HAdV-14p1. This deletion, unique among HAdV B2 strains, corresponds to amino acid residues lysine and glutamic acid (AAA/GAA) at codon positions 251/252 located in the F–G loop of the fiber protein knob. Other than this deletion, the fiber sequences were highly conserved and had 99.3% nt identity to the HAdV-14p de Wit type strain, 99.1% identity to the 11a strains, 99% identity to the Spain 11a/14 strain in 1969, and only 93%–94% similarity to the HAdV-11p strains. The de Wit prototype strain had 99%–99.1% identity to the Spain 11/14 strain 273 from 1969 and other 11a strains, which have been identified as intertypic recombinants with a HAdV-11 hexon gene and a HAdV-14-like fiber gene (*7*).

The 870 bp of the E1A ORF from the Ireland sequences had higher sequence identity to HAdV-11a strains (99.8%) and the prototype HAdV-14p de Wit strain (99.1%) than to HAdV-11p strains (97%) or any other HAdV B2 reference strains. Furthermore, the Ireland E1A sequences contain a 3-nt insertion, GTG, which is also present in all of the 11a and 14p1 strains but not in the 14p strains. The HAdV-14p1 E1A sequences were most closely associated with the 11a sequences from Taiwan, Singapore, Spain, and the United States, and the fiber sequences from Ireland clustered close to the 11a and 14p sequences. The hexon ORFs analyzed from the samples from Ireland were 2,838 nt and were almost identical (99.86%) to the prototype HAdV-14p de Wit strain. Compared with the E1A and fiber gene sequences, in the hexon region the Ireland sequences exhibited much lower sequence identity to the HAdV-11a and -11p strains analyzed in the study; the HAdV-11a and -11p hexon genes were much more similar to each other and branched separately on the tree (Figure 1, panel C). Viral DNA extracted from each of the 8 isolates of HAdV-14 yielded profiles identical to each other and identical to those reported for the North American strain of HAdV-14. The Ireland isolates were therefore identified as corresponding to genome type 14p1 on the basis of the distinct *Bcl*I, *Bst*EII, and *Pst*I restriction patterns (Figure 2).

**Figure 2:** Comparative restriction enzyme analysis of viral DNA extracted from the prototype human adenovirus (HAdV) 14 de Wit strain and the first detected HAdV-14 case isolated in Dublin, Ireland, November 2009. All odd-numbered lanes (e.g., 1, 3) contain the de Wit strain and all even-numbered lanes (e.g., 2, 4) contain the Dublin 2009 strain, with restriction enzyme digests as follows: lanes 1 and 2 with *BamH*I; lanes 3 and 4 with *Bcl*I; lanes 5 and 6 with *Bgl*II; lanes 7 and 8 with *BstE*II; lanes 9 and 10 with *Dra*I; lanes 11 and 12 with *Hind*III; lanes 13 and 14 with *Pst*I; lanes 15 and 16 with *Sma*I; lanes 17 and 18 with *Xba*I. Outer lanes are molecular markers (1 Kb +100 bp; BioRad, Hercules, CA, USA).

Of the 9 HAdV-14p1–infected patients, 8 (90%) had respiratory symptoms: pneumonia was diagnosed for 4, bronchiolitis for 3, and acute respiratory distress syndrome (ARDS) for 1. The other patient (case-patient 4; a premature infant born at 35 weeks) was hypotonic at birth with abnormal liver function (Table). Major underlying medical conditions were reported in 4 patients: a BMT recipient; a premature neonate; a patient co-infected with HIV and hepatitis C virus; and a patient with the craniofacial abnormality Pierre Robin syndrome, who had a prior tracheostomy and percutaneous enteral gastrostomy. ARDS developed in the BMT recipient (case-patient 6) 56 days posttransplant; lymphocyte count had not recovered and was at undetectable levels (<0.3/mm^3^). The patient co-infected with HIV and hepatitis C virus was receiving antiretroviral therapy and had undetectable HIV RNA (<50 copies/mL), a CD4 count of 436 cells/mL (15%), and a resolved hepatitis C virus infection. No underlying conditions were reported for the 5 remaining patients. Three patients died in May 2010: the premature neonate, the BMT recipient, and a 46-year-old woman. The woman had no known notable medical history but had a high body mass index and smoked cigarettes (Table). Two patients (case-patients 4 and 8) received extracorporeal membrane oxygenation. Case-patient 4 had a natural killer (NK) cell deficiency and died. The nature of the NK cell defect in the premature infant was not defined, and bone marrow was analyzed with no pathologic findings noted. Leukocyte screen, however, showed a lack of NK cells; additional screen for immunodeficiencies (such as severe combined immunodeficiency) proved inconclusive, and a human leukocyte antigen–B57–negative haplotype was recorded. Case-patient 8, however, for whom ARDS had been diagnosed, received prolonged (>6 weeks) extracorporeal membrane oxygenation and recovered. Four patients had an unremarkable full recovery.

Of the 9 patients positive for Ireland HAdV-14p1, serum or plasma samples of 2 were submitted for HAdV DNA quantification. The preterm infant (case-patient 4) had a HAdV load in serum of 8.18 log_10_ viral genomes/mL, and the infant with ARDS (case-patient 8) had a plasma adenoviral load of 8.83 log_10_ viral genomes/mL. The immunocompetent 46-year-old woman (case-patient 5) had a viral load of 4.80 log_10_ genomes/g from a postmortem lung biopsy specimen.

## Discussion

We have demonstrated that the HAdV-14p1 strain first identified in the United States is now circulating in Ireland and is associated with substantial illness and with 3 deaths: a BMT recipient, a neonate, and (most notably) an immunocompetent, apparently otherwise healthy adult. The role of HAdV-14p1 in the deaths remains to be elucidated because 2 of the 3 deaths occurred in substantially immunocompromised patients. Conversely, other patients who were also immunocompromised recovered unremarkably, and fatal severe pneumonia and ARDS are known to occur in immunocompetent adults infected with other HAdV serotypes (*24*). Nevertheless, of concern in the cohort described here is the third death, which occurred in a previously well patient. The immunocompetent woman was a smoker, and smoking has been identified as an independent risk factor possibly facilitating transmission of HAdV-14p1 (*11*). However, the low rate of infections (5%) observed by Esposito et al. (*11*) in household contacts of persons infected with the emerging virus suggests that infection is unlikely to spread in the community. Efficient transmission of HAdV-14p1 appears to require close physical contact, as supported by the observation that in barracked communities used for military trainees in the United States, antibody titers demonstrated recent exposure after entry to the facility (*25*). Of interest in the HAdV-14p1 cases identified in the present study is the higher frequency of male patients (78%) infected; this observation has been reported by other groups (*19**,**26*), although the numbers identified in our study are insufficient to make definitive conclusions. Previous reports have noted that prior vaccination with HAdV-7, a subspecies B1 virus, of military recruits may confer cross-protection, presumably from heterotypic immunity, and that this vaccine could potentially be used to reduce HAdV-14p1 disease severity (*27**,**28*). Trei et al. have reported on the spread of HAdV-14 in 2007 from a large military training facility in Texas to secondary sites across the United States and to South Korea, despite the institution of active surveillance for acute respiratory disease and prevention and control measures (*29*). Despite evidence of decreased spread from military personnel to family members outside the training population, many recent military graduates may have been asymptomatic and incubating the virus, which presents a possible scenario for the subsequent dissemination of this virus.

Our findings suggest that clinicians and other health care workers should consider HAdV-14p1 in the differential diagnosis of community-acquired pneumonia. No evidence indicates that HAdV-14p1 infection is substantially more severe than that by other HAdV serotypes; however, we propose increased surveillance for this emerging agent in Europe and elsewhere in immunologically naive populations. In addition, we suggest the retrospective investigation of HAdV-positive specimens for HAdV-14p1, particularly from untyped viruses from patients who had severe disease and who died without an established etiology to help determine the time and location of reemergence of this HAdV in Europe.

## References

[1] Rowe WP, Huebner RJ, Gilmore LK, Parrott RH, Ward TJ. Isolation of a cytopathogenic agent from human adenoids undergoing spontaneous degeneration in tissue culture. Proc Soc Exp Biol Med. 1953;84:570–3.1313421710.3181/00379727-84-20714

[2] Hilleman MR. Recovery of new agent from patients with acute respiratory illness. Proc Soc Exp Biol Med. 1954;85:183–8.1313432910.3181/00379727-85-20825

[3] Wold WSM, Horwitz MS. Adenoviruses. In: Fields BN, Knipe DM, Howley PM, editors. Fields virology. 5th ed. Philadelphia (PA): Lippincott-Raven; 2007. p. 2395–436.

[4] Baum SG. Adenoviruses. In: Mandell GL, Bennett JE, Dolin R. editors. Principles and practice of infectious diseases. 5th ed. Vol 2. Philadelphia (PA): Churchill Livingstone; 2005. p. 1835–41.

[5] Van Der Veen J, Kok G. Isolation and typing of adenoviruses recovered from military recruits with acute respiratory disease in the Netherlands. Am J Hyg. 1957;65:119–29.1341090410.1093/oxfordjournals.aje.a119860

[6] Kendall EJ, Riddle RW, Tuck HA, Rodan KS, Andrews BE, McDonald JC. Pharyngo-conjunctival fever; school outbreaks in England during the summer of 1955 associated with adenovirus types 3, 7, and 14. BMJ. 1957;2:131–6. 10.1136/bmj.2.5037.13113436877PMC1961818

[7] Kajon AE, Lu X, Erdman DD, Louie J, Schnurr D, George KS, Molecular epidemiology and brief history of emerging adenovirus 14–associated respiratory disease in the United States. J Infect Dis. 2010;202:93–103. 10.1086/65308320500088

[8] Metzgar D, Osuna M, Kajon AE, Hawksworth AW, Irvine M, Russell KL. Abrupt emergence of diverse species B adenoviruses at US military recruit training centers. J Infect Dis. 2007;196:1465–73. 10.1086/52297018008225

[9] Centers for Disease Control and Prevention. Acute respiratory disease associated with adenovirus serotype 14—four states, 2006–2007. MMWR Morb Mortal Wkly Rep. 2007;56:1181–4.18004235

[10] Louie JK, Kajon AE, Holodniy M, Guardia-LaBar L, Lee B, Petru AM, Severe pneumonia due to adenovirus serotype 14: a new respiratory threat? Clin Infect Dis. 2008;46:421–5. 10.1086/52526118173356

[11] Esposito DH, Gardner TJ, Schneider E, Stockman LJ, Tate JE, Panozzo CA, Outbreak of pneumonia associated with emergent human adenovirus serotype 14—southeast Alaska, 2008. J Infect Dis. 2010;202:214–22. 10.1086/65349820533881

[12] Wang H, Tuve S, Erdman DD, Lieber A. Receptor usage of a newly emergent adenovirus type 14. Virology. 2009;387:436–41. 10.1016/j.virol.2009.02.03419307010PMC2674115

[13] Houng HS, Gong H, Kajon AE, Jones MS, Kuschner RA, Lyons A, Genome sequences of human adenovirus 14 isolates from mild respiratory cases and a fatal pneumonia, isolated during 2006–2007 epidemics in North America. Respir Res. 2010;11:116. 10.1186/1465-9921-11-11620738863PMC2933684

[14] Hierholzer JC, Pumarola A. Antigenic characterization of intermediate adenovirus 14–11 strains associated with upper respiratory illness in a military camp. Infect Immun. 1976;13:354–9.17736510.1128/iai.13.2.354-359.1976PMC420619

[15] Chmielewicz B, Benzler J, Pauli G, Krause G, Bergmann F, Schweiger B. Respiratory disease caused by a species B2 adenovirus in a military camp in Turkey. J Med Virol. 2005;77:232–7. 10.1002/jmv.2044116121380PMC7166463

[16] Kajon AE, Erdman DD. Assessment of genetic variability among subspecies B1 human adenoviruses for molecular epidemiology studies. In: Wold W, Tollefsson A, editors. Adenovirus methods and protocols. 2nd ed. Vol 2. Totowa (NJ): Humana Press; 2007. p. 335–55.10.1007/978-1-59745-277-9_2317656793

[17] Treacy A, Carr MJ, Dunford L, Palacios G, Cannon GA, O’Grady A, First report of sudden death due to myocarditis caused by adenovirus serotype 3. J Clin Microbiol. 2010;48:642–5. 10.1128/JCM.00815-0919940052PMC2815630

[18] Casas I, Avellon A, Mosquera M, Jabado O, Echevarria JE, Campos RH, Molecular identification of adenoviruses in clinical samples by analyzing a partial hexon genomic region. J Clin Microbiol. 2005;43:6176–82. 10.1128/JCM.43.12.6176-6182.200516333124PMC1317187

[19] Lewis PF, Schmidt MA, Lu X, Erdman DD, Campbell M, Thomas A, A community-based outbreak of severe respiratory illness caused by human adenovirus serotype 14. J Infect Dis. 2009;199:1427–34. 10.1086/59852119351259

[20] Burland TG. DNASTAR's Lasergene sequence analysis software. Methods Mol Biol. 2000;132:71–91.1054783210.1385/1-59259-192-2:71

[21] Thompson JD, Higgins DG, Gibson TJ. CLUSTAL W: improving the sensitivity of progressive multiple sequence alignment through sequence weighting, position-specific gap penalties and weight matrix choice. Nucleic Acids Res. 1994;22:4673–80. 10.1093/nar/22.22.46737984417PMC308517

[22] Hall TA. BioEdit: a user-friendly biological sequence alignment editor and analysis program for Windows 95/98/NT. Nucleic Acids Symp Ser. 1999;41:95–8.

[23] Swofford DL. PAUP*: phylogenetic analysis using parsimony (*and other methods), version 4.0 β 10. Sunderland (MA): Sinauer Associates; 2003.

[24] Hakim FA, Tleyjeh IM. Severe adenovirus pneumonia in immunocompetent adults: a case report and review of the literature. Eur J Clin Microbiol Infect Dis. 2008;27:153–8. 10.1007/s10096-007-0416-z18030505PMC7101838

[25] Tate JE, Bunning ML, Lott L, Lu X, Su J, Metzgar D, Outbreak of severe respiratory disease associated with emergent human adenovirus serotype 14 at a US Air Force training facility in 2007. J Infect Dis. 2009;199:1419–26. 10.1086/59852019351260

[26] Centers for Disease Control and Prevention. Outbreak of adenovirus 14 respiratory illness—Prince of Wales Island, Alaska, 2008. MMWR Morb Mortal Wkly Rep. 2010;59:6–10.20075838

[27] Lyons A, Longfield J, Kuschner R, Straight T, Binn L, Seriwatana J, A double-blind, placebo-controlled study of the safety and immunogenicity of live, oral type 4 and type 7 adenovirus vaccines in adults. Vaccine. 2008;26:2890–8. 10.1016/j.vaccine.2008.03.03718448211

[28] Russell KL, Hawksworth AW, Ryan MA, Strickler J, Irvine M, Hansen CJ, Vaccine-preventable adenoviral respiratory illness in US military recruits, 1999–2004. Vaccine. 2006;24:2835–42. 10.1016/j.vaccine.2005.12.06216480793PMC1955759

[29] Trei JS, Johns NM, Garner JL, Noel LB, Ortman BV, Ensz KL, Spread of adenovirus to geographically dispersed military installations, May–October 2007. Emerg Infect Dis. 2010;16:769–75.2040936510.3201/eid1605.091633PMC2954003

# Research article: Deaths Associated with Human Adenovirus-14p1 Infections, Europe, 2009–2010

**DOI:** 10.3201/1708.101760

**Authors:** Michael J. Carr, Adriana E. Kajon, Xiaoyan Lu, Linda Dunford, Paul O’Reilly, Paul Holder, Cillian F. De Gascun, Suzie Coughlan, Jeff Connell, Dean D. Erdman, William W. Hall

**Affiliations:** Author affiliations: National Virus Reference Laboratory–University College Dublin, Dublin, Ireland (M.J. Carr, L. Dunford, P. O’Reilly, P. Holder, C.F. De Gascun, S. Coughlan, J. Connell, W.W. Hall);; Lovelace Respiratory Research Institute, Albuquerque, New Mexico, USA (A.E. Kajon);; Centers for Disease Control and Prevention, Atlanta, Georgia, USA (X. Lu, D.D. Erdman)

## Earning CME Credit

To obtain credit, you should first read the journal article. After reading the article, you should be able to answer the following, related, multiple-choice questions. To complete the questions (with a minimum 70% passing score) and earn continuing medical education (CME) credit, please go to **www.medscape.org/journal/eid**. Credit cannot be obtained for tests completed on paper, although you may use the worksheet below to keep a record of your answers. You must be a registered user on Medscape.org. If you are not registered on Medscape.org, please click on the New Users: Free Registration link on the left hand side of the website to register. Only one answer is correct for each question. Once you successfully answer all post-test questions you will be able to view and/or print your certificate. For questions regarding the content of this activity, contact the accredited provider, CME@medscape.net. For technical assistance, contact CME@webmd.net. American Medical Association’s Physician’s Recognition Award (AMA PRA) credits are accepted in the US as evidence of participation in CME activities. For further information on this award, please refer to http://www.ama-assn.org/ama/pub/category/2922.html. The AMA has determined that physicians not licensed in the US who participate in this CME activity are eligible for *AMA PRA Category 1 Credits*™. Through agreements that the AMA has made with agencies in some countries, AMA PRA credit may be acceptable as evidence of participation in CME activities. If you are not licensed in the US, please complete the questions online, print the certificate and present it to your national medical association for review.

## Article Title: Deaths Associated with Human Adenovirus-14p1 Infections, Europe, 2009–2010

## CME Questions

**1. Based on the above case series by Dr. Carr and colleagues, which of the following statements about demographic, genetic, and transmission-related factors associated with human adenovirus-14p1 (HAdV-14p1) infections recently detected in Ireland is most likely correct?**A. These HAdV-14p1 variants are phylogenetically different from those previously detected in the United StatesB. Most of the patients were young womenC. Geographic clustering was observedD. Efficient transmission of HAdV-14p1 appears to require close physical contact**2. You are a public health official asked to consult with local healthcare facilities in a European region where HAdV-14p1 infections have been detected. Based on the above study, which of the following statements about anticipated clinical characteristics and complications associated with HAdV-14p1 infections is most likely to appear in your report?**A. Initial presentation is usually with gastrointestinal symptomsB. A very small proportion of patients are likely to have significant comorbidityC. A little less than half of patients are likely to have an unremarkable full recoveryD. No death has been reported in an immunocompetent individual**3. As the public health official described in question 2, which of the following recommendations would you be most likely to make in your report, based on the clinical and public health implications of the case series described above?**A. Surveillance for HAdV-14p1 should remain the sameB. Healthcare workers should be advised that HAdV-14p1 infection is significantly more severe than that of other HAdV serotypesC. HAdV-positive specimens should be retrospectively investigated for HAdV-14p1, particularly from untyped viruses from patients who had severe or fatal disease without established etiologyD. HAdV-14p1 should not be included in the differential diagnosis of community-acquired pneumonia

## Activity Evaluation

**Table:** 

1. The activity supported the learning objectives.				
Strongly Disagree				Strongly Agree
1	2	3	4	5
2. The material was organized clearly for learning to occur.				
Strongly Disagree				Strongly Agree
1	2	3	4	5
3. The content learned from this activity will impact my practice.				
Strongly Disagree				Strongly Agree
1	2	3	4	5
4. The activity was presented objectively and free of commercial bias.				
Strongly Disagree				Strongly Agree
1	2	3	4	5
